# Analysis of the Leap Motion Controller’s Performance in Measuring Wrist Rehabilitation Tasks Using an Industrial Robot Arm Reference

**DOI:** 10.3390/s22134880

**Published:** 2022-06-28

**Authors:** Rogério S. Gonçalves, Marcus R. S. B. de Souza, Giuseppe Carbone

**Affiliations:** 1 School of Mechanical Engineering, Federal University of Uberlândia, Uberlândia 38408-016, Brazil; 2 SENAI, Serra 29173-087, Brazil; mrsbs.mecatronica@gmail.com; 3 Department of Mechanical, Energy and Management Engineering, University of Calabria, 87036 Rende, Italy; giuseppe.carbone@unical.it

**Keywords:** leap motion controller, hand, wrist, accuracy, repeatability

## Abstract

The Leap Motion Controller (LMC) is a low-cost markerless optical sensor that performs measurements of various parameters of the hands that has been investigated for a wide range of different applications. Research attention still needs to focus on the evaluation of its precision and accuracy to fully understand its limitations and widen its range of applications. This paper presents the experimental validation of the LMC device to verify the feasibility of its use in assessing and tailoring wrist rehabilitation therapy for the treatment of physical disabilities through continuous exercises and integration with serious gaming environments. An experimental set up and analysis is proposed using an industrial robot as motion reference. The high repeatability of the selected robot is used for comparisons with the measurements obtained via a leap motion controller while performing the basic movements needed for rehabilitation exercises of the human wrist. Experimental tests are analyzed and discussed to demonstrate the feasibility of using the leap motion controller for wrist rehabilitation.

## 1. Introduction

The wrist is one of the most important joints of the human body. Through gripping, lifting, twisting, and bending the wrist makes most of the possible daily activities. Unfortunately, the wrists are often impaired by nagging pain and stiffness. Wrist injuries and disorders are often caused by sprains, fractures, repetitive stress, carpal tunnel syndrome, arthritis, ganglion cysts, Kienbock’s disease, tendinitis, and stroke [1]. Often, wrist tension can be caused by a limited range of motion plus a lack of blood flow to the joints. Pain and impairment can also be symptomatic of a more serious condition, such as carpal tunnel syndrome, arthritis, and wrist sprain or strain. Due to the wrists’ heavy involvement in most daily activities, stiffness and pain can affect athletes and office workers alike. Wrist injuries are especially common in professions and athletes that require heavy or repetitive use of the hands and wrists. Approximately 25% of all sports-related injuries involve the hand or wrist [2]. Stroke is a leading cause of long-term disability and leaves a considerable number of individuals with motor deficits. One consequence of stroke is wrist spasticity [3,4]. Spasticity is a muscular disorder characterized by muscle tightness and stiffness, which can affect muscles throughout the body, including the hand, and can be painful.

Rehabilitation training is one effective way to reduce impairment and pain for most human wrist injuries and disorders, as mentioned, for example, in [5]. Together with the rehabilitation procedure, it is often necessary to quantify the evolution of the treatment. This often requires the measurement of the Range Of Motion (ROM) that is essential for the development of effective rehabilitation protocols. Currently, the clinical standard is based on using a goniometer [6]. Wrist rehabilitation therapy involves monotonous and repetitive movements/exercises [7], and serious games can be an option to make an interactive rehabilitation experience and generate more motivation in patients. Along with serious games, some devices are desirable to acquire and store movement data and, if possible, use them interactively with the patient.

One device that permits virtual interactions with hands is the Leap Motion Controller (LMC). The Leap Motion Controller is a gesture sensor used to interact with a computer, which uses infrared sensors to collect data about the position and motions of a user’s hands. The users do not need other sensors/devices coupled with their bodies. This sensor has been preliminarily used in combination with serious games for hand rehabilitation, such as outlined in [8,9,10,11,12]. In [13], the authors conducted a review of the use of LMC as a tool in the treatment of the upper limb in people with stroke and concluded the necessity of future research protocols with greater scientific rigor. Though the researchers developed serious games for hand/wrist rehabilitation, the applications lacked reliable information in terms of the functions of the different disposition of the fingers in the hand, that is, the functions of the specific injuries and disorders. It is necessary, also, to evaluate the accuracy of LMC in the specific configuration of the physiotherapist’s exercises and data collected. Therefore, in this paper, we propose a detailed analysis of LMC when used for measuring hand/wrist poses in typical rehabilitation tasks. The proposed analysis is based on a specific experimental set up that uses a high accuracy industrial robot as pose reference.

This paper is structured as follows: Section 2 provides a focused review on the specifications for a LMC to wrist/hand tracking, followed by a brief review on the kinesiology, injuries/disorders, and rehabilitation procedures of the human wrist in Section 3. The experimental set up and methodology of the experiments are presented in Section 4. The detailed results are analyzed and discussed in Section 5. Finally, the conclusions and recommendations are outlined in Section 6.

## 2. Leap Motion Controller

**Figure 1:**
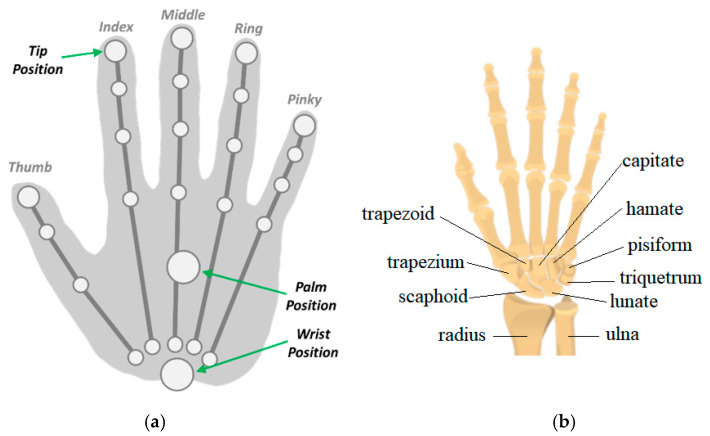
Entities belonging to the hand class. (**a**) Leap Motion tracking points. (**b**) Wrist and finger bones.

The LMC is a low-cost, off-the-shelf product that costs an average of USD 100; it consists of two cameras and three infrared LEDs. Its operation is based on the principle of stereoscopy, and the images obtained by the cameras are recorded in its internal memory to be later transmitted via USB interface to the tracking software [14,15]. The images collected by the LMC, after processing, give information of the hand, such as position and orientation Cartesian coordinates of the fingertips, palm, and wrist of the hand, for example [14]. The processing of the LMC data taken from the images is performed by the Application Programmer Interface (API) that provides a set of functions that can be accessed to obtain the parameters calculated from the sensor. The data acquired by the LMC are made available by the API through data structures called frames. Each frame is generated from the images collected by the LMC in the current iteration and contains information about the tracked entities such as hands, fingers, wrist position, and finger bones. The frame structure named “hand class” has attributes such as the direction vectors and handgrip angle, the position and speed coordinates of the palm, Figure 1a.

In [14], a pen attached to an industrial robotic was used as a reference standard system for LMC sensor validation. The error calculated for the LMC readings in the static tests was less than 0.2 mm, regardless of the axis analyzed, and in the dynamic linear paths it was 1.2 mm on average. The repeatability observed in the static tests was less than 0.17 mm for the *x* axis and in the dynamic tests 0.4 mm on average. In [16], the accuracy, reliability, and sample rate of the LMC were evaluated using camera tracking was used as a reference system. In static experiments, a plastic hand attached to support was used to simulate the human hand in 37 distinct positions within the LMC workspace. The results showed a drop in the accuracy of the measurements as the tool moved away from the sensor. In the experiments, the sample rate proved to be quite inconsistent, presenting an average of 39 Hz with a standard deviation of 12.8 Hz.

In [17], a system where a robotic arm reproduced the movements of a human hand, represented by a metal rod 7 mm in diameter, in real-time was proposed. The authors evaluated the accuracy of the LMC using the robotic arm as a reference system to make static and dynamic tests. The error measured in the static experiments was less than 0.01 mm when the metal rod was close to the origin of the LMC, and the repeatability was 1 mm. The authors observed that the repeatability worsens as the tool moves away from the sensor. In [18], the accuracy of the LMC when measuring the distance between the tip of the index finger and the thumb when the hand is in a clamp configuration was evaluated. Distances of 10 to 130 mm were used between the fingertips in the tests, and, for each distance, 20 measurements were made. It was found that the general mean quadratic error (RMS) was 4.44 mm, having been lower for greater distances. In [19], three healthy individuals participated to take data from hand using the LMC. The average difference between a gold-standard reference device (0.1 mm) and the LMC for pinch distance was −0.86 ± 10.8 mm.

In [15], the average and maximum errors observed in the static tests were 17.47 and 33.65 mm, respectively. It was concluded that the error tends to increase as the hand moves away from the sensor. The average value for repeatability was 0.25 mm, indicating good accuracy in repeatability conditions. In [20], the ability of the LMC to track the flexion/extension, abduction/adduction movements of the wrist, and pronation/supination of the forearm was evaluated. For validation, a motion capture system with markers from Motion Analysis Corporation was used. The results of the experiments showed that the mean quadratic errors were 11.6° for the flexion/extension movements, 12.4° for abduction and adduction, and 38.4° for supination/pronation. The authors concluded that the LMC can provide satisfactory measures for the flexion/extension and abduction/adduction movements. In [6], the ability of the LMC to measure the range of hand and wrist movements of 20 healthy volunteers was explored. A goniometer was used as a comparison; the results were flexion (9°)/extension (3°), pronation (13°)/supination (39°), and abduction (7°)/adduction (3°). The values in parentheses represent the mean absolute difference between the measurements.

Several papers [14,15,16,17] were presented on the analysis of the precision and accuracy of the LMC almost static measurement with a tool simulating the hand. The angular displacement capacity of LMC to measure the wrist movements is little explored in the literature and without considering different dispositions of the fingers about the palm used in wrist rehabilitation procedures.

## 3. Wrist, Injuries, and Rehabilitation

The wrist connects the hand to the forearm and has several small joints. This makes it flexible to move the hand in diverse ways. The wrist has two large forearm bones and eight small bones named carpals, Figure 1b. It also has tendons, which connect muscles, and ligaments that connect bones [1]. The wrist can be considered to be a mechanical spherical joint [21].

Wrist injuries and disorders are often caused by sprains, fractures, repetitive stress, carpal tunnel syndrome, arthritis, ganglion cysts, Kienbock’s disease, tendinitis, and stroke. Sudden impacts are mainly responsible for wrist sprains (injuries in ligaments, muscles, or tendons) and fractures (broken bones). Repetitive activities that involve wrist motion, such as some kind of sports that hit balls, can inflame the tissues around joints and cause stress. Wrist arthritis is the loss of cartilage between the wrist bones; it causes pain when the patient is turning the hand palm up or palm down. Carpal tunnel syndrome is increased pressure on the median nerve, which passes through the carpal tunnel and causes pain, numbness, and tingling. Ganglion cysts may be painful and are soft tissue cyst that occurs most often on the part of the writs opposite palm. The pain may either worsen or improve with activity. Kienbock’s disease is a progressive collapse of the small lunate bone in the wrist and occurs when the blood supply to the bone is compromised. This disease can lead to progressive wrist pain and abnormal carpal motion. Tendonitis is an inflammation of a tendon, usually due to overuse.

One wrist problem is a function of the stroke or brain injury that can cause spasticity [22,23,24]. Spasticity is a muscular disorder characterized by muscle tightness and stiffness, which can affect muscles throughout the body including the hand and can be painful. About 30% of stroke survivors will have spasticity. When a stroke occurs, the nervous system sustains damage and can disrupt the signals between the brain and muscles. As a result, certain muscles can become over-active and are unable to relax, leaving them in a state of prolonged and involuntary contraction. The hand and fingers are stiff and hard to move. In the case of severe spasticity, the person’s fingers may curl, and their hand may remain clenched in a fist [24]. If the spasticity is not treated, it can progress into contractures, i.e., when the connective tissue and joints become extremely stiff, often painful, and limit the range of motion. These contractures make the actions of important daily tasks difficult [4].

### Wrist/Hand Rehabilitation Exercises

After wrist injuries, it is necessary to complete rehabilitation exercises if pain allows. Rehabilitation exercises can improve mobility and strengthen the muscles in the wrist, hand, and forearm [25]. In some cases, the exercises are made with open fingers or closed fingers.

**Figure 2:**
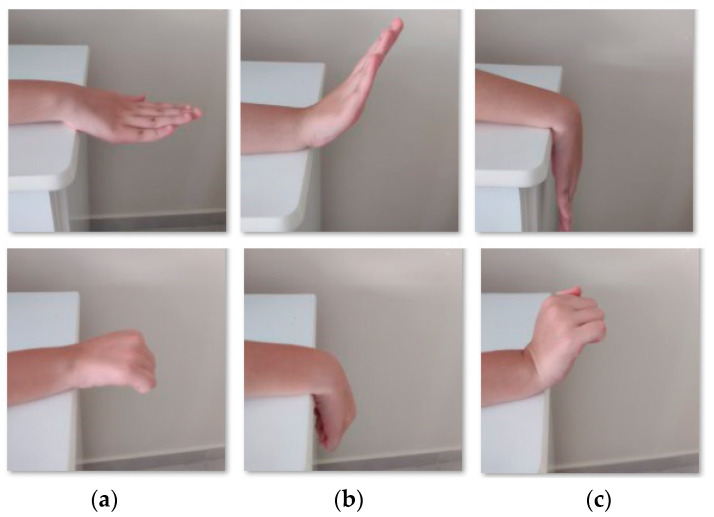
Examples of wrist flexion/extension with open and close fingers. (**a**) Resting position. (**b**) Flexion movement. (**c**) Extension movement.

The wrist flexion/extension exercise [21], Figure 2, starts with the forearm resting on support and hand hanging off the table, Figure 2a. After, it is necessary to bend the wrist down, i.e., the extension movement, and hold it for 5 s, Figure 2b. The next step is to return slowly to the resting position. The wrist is then bent up making the flexion movement, Figure 2c, and held for 5 s, before slowly returning to the starting position.

**Figure 3:**
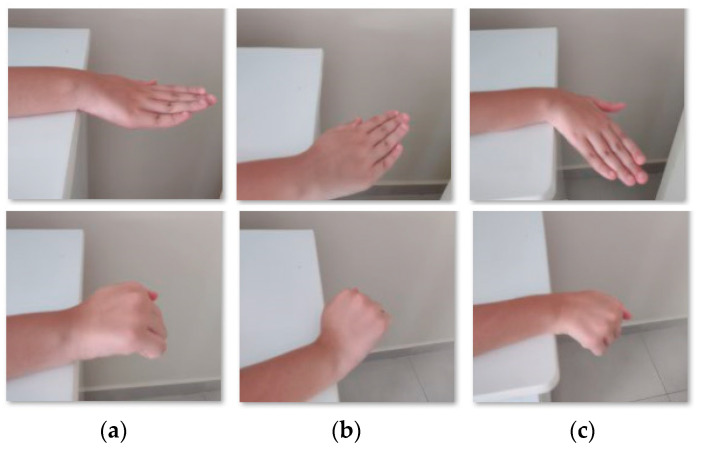
Examples of wrist deviations with open and close fingers. (**a**) Resting position. (**b**) Radial deviation. (**c**) Ulnar deviation.

The wrist deviation movements [21] need the forearm resting on a support and the hand hanging off the support, Figure 3a. The movements start slowly by turning the hand to the side, Figure 3b, and holding for 5 s and after returning slowly to starting position. In the next step, the patient turns the hand to another side, Figure 3c, holds for 5 s, and returns to starting position.

**Figure 4:**
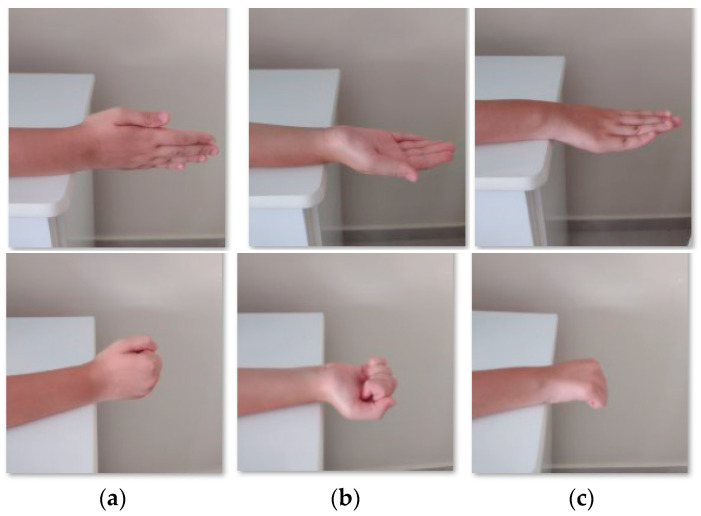
Examples of supination/pronation with open and close fingers: (**a**) resting position, (**b**) supination, and (**c**) pronation.

**Figure 5:**
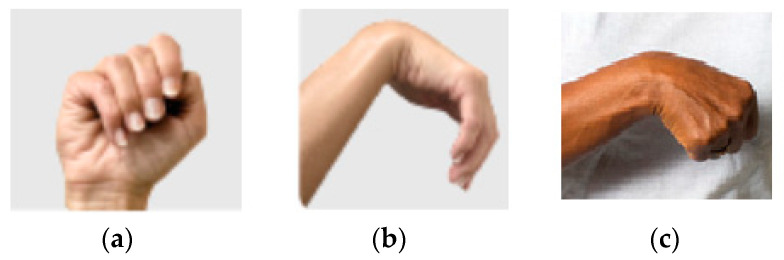
Examples of hand spasticity: (**a**) clenched fist, (**b**) flexed wrist, and (**c**) bent wrist.

The supination/pronation corresponds to the rotation of the forearm [21]. The movement starts with resting the forearm next to the body, palm out, Figure 4a. The patient then turns the hands up, Figure 4b, and holds for 5 s. After slowly turning the hand down, the patient returns it to the initial position and holds for 5 s. Then, the patient turns the hand in the opposite direction, Figure 4c, and holds for 5 s before returning to the initial position. The wrist tendonitis rehabilitation exercises involve the base movements of the wrist: flexion/extension, supination/pronation, and radial/ulnar deviation. The spasticity, Figure 5, can be treatable by rehabilitation exercises [24].

Rehabilitation exercises are one of the most effective ways to treat the spasticity in the hand. Therapeutic movements of the hand can help the neuroplasticity, i.e., the nervous system rewiring, and create new pathways between the brain and muscles. This process needs time and effort to make repetitive practice/movements. The spasticity is velocity-dependent; if the hand moves faster, the affected muscles are stronger contract. Therefore, is important to stretch the hand slowly. If the fingers are affected by spasticity is important to keep the wrist position, because the muscles groups that are primarily responsible for flexing and extending the fingers are in the forearm with tendons crossing the wrist connecting the finger bones. The exercises to decrease wrist/hand stiffness involve pronation/supination, flexion/extension of the wrist, and finger flexion/extension.

## 4. Methodology of Experiments

**Figure 6:**
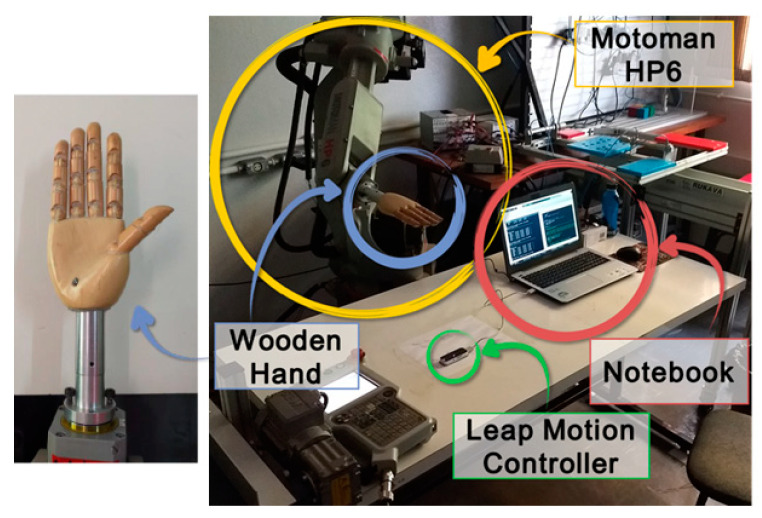
The proposed experimental apparatus.

We identified an industrial robot with certified positioning repeatability higher than 0.1 mm as our gold-standard reference for our motion analysis tests. Namely, to simulate the hand movements, a Yaskawa industrial robot model Motoman HP6, NX100 controller was used, with an anthropomorphic and anthropometric right-hand model like an end effector for precise angular movements, during the experiments. The robot has a repeatability of 0.08 mm. The LMC was fixed to a table in front of the robotic arm so that the wooden hand attached to the robot could move in different orientations within the sensor’s workspace. The disposition of the devices in the experimental apparatus, as well as the wooden hand model used, are shown in Figure 6. 

**Figure 7:**
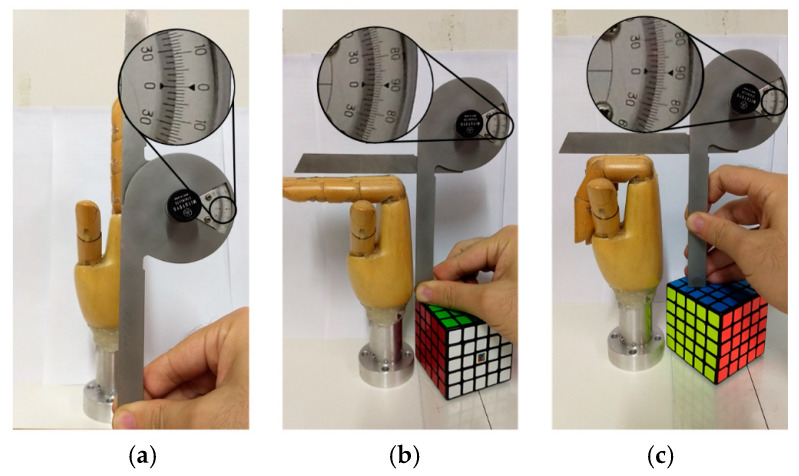
Hand configurations: (**a**) open, (**b**) folded, and (**c**) closed.

**Figure 8:**
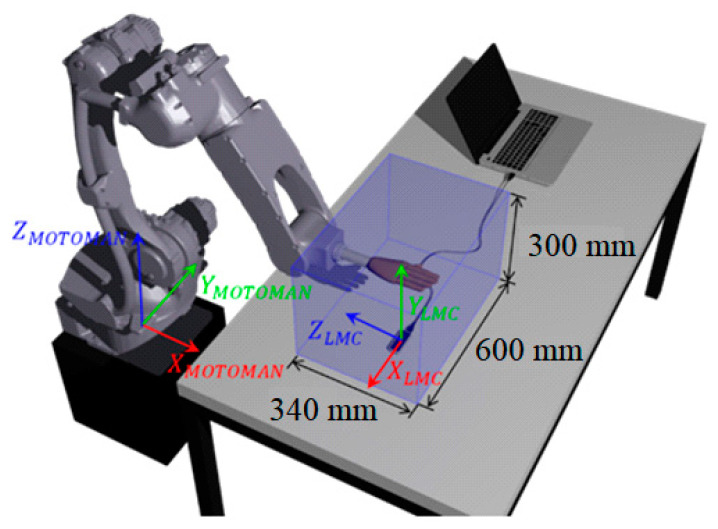
Cartesian axes of the robotic arm and workspace were used in the experiments.

Three wooden hand model configurations were used, with the open hand, Figure 7a, with the fingers bent at 90° about the longitudinal axis of the palm, Figure 7b, and with the closed hand, Figure 7c, with the fingers at 175°. In all configurations, the thumb is apart, and the other fingers are close to each other. These settings aim to replicate the possible positions in the rehabilitation exercises, Figure 2, Figure 3, Figure 4 and Figure 5. A workspace was defined to avoid collisions between the sensor, table, and laptop during the industrial robot’s movements, as shown in Figure 8. That workspace is inside of the LMC workspace.

The Cartesian reference system used originates from the center of LMC upper surface and orientation as shown in Figure 8. The data acquisition was made using a developed Matlab code on a laptop (Intel^®^ Core i7-6500U 2.5 GHz 8 GB) with Windows 10 and the LMC API in version V3. The communication between the LMC API and Matlab was made using an adaptation of the MatLeap library [26].

Angular experiments, in each of the three axes of the LMC, Figure 8, were realized to compared with the literature and insert new configurations not analyzed.

In Orion version 3 of the API, used in this paper, it is not possible to track tools such as pens or rods [15]. Thus, to validate the measurements, one point in the hand was used, since the hand model is considered rigid. The sensor evaluation was performed by calculating the systematic error and its precision (repeatability) relative to the predetermined reference position in each experiment. The estimation of these parameters was made according to the ISO 9283 standard [27], concerning the performance evaluation of industrial manipulators.

### 4.1. Analysis of Orientation Measures

**Figure 9:**
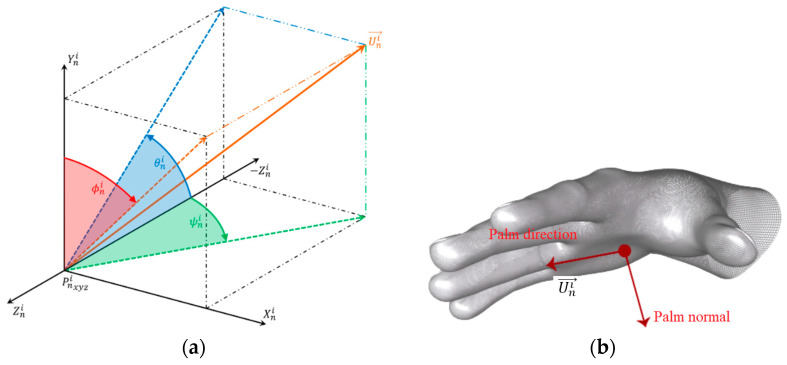
(**a**) Spatial representation of angles $\theta_{n}^{i}$, $\psi_{n}^{i}$, and $\phi_{n}^{i}$ in relation to the unit direction vector $\vec{U_{n}^{i}}$. (**b**) Vector palm direction ($\vec{U_{n}^{i}}$) calculated by the LMC.

The analysis of the orientation measurements was made using the data output of the LMC sensor in terms of the vector palm direction vector ($\vec{U_{n}^{i}}$) versus time. The direction of the vector ($\vec{U_{n}^{i}}$) relative to the hand is shown in Figure 9b. For the analysis of the orientation measurements, the angles were considered to be $\theta_{n}^{i}$, which symbolizes a rotation of the direction vector $\vec{U_{n}^{i}}$ on the *x* axis and is equivalent to the angle between the negative *z* axis and the projection of this vector on the *y*–*z* plane. The angle $\psi_{n}^{i}$ corresponds to a rotation of the direction vector $\vec{U_{n}^{i}}$ on the *y* axis and is equivalent to the angle between the negative *z* axis and the projection of this vector on the *x*–*z* plane. The angle $\phi_{n}^{i}$ represents a rotation of the direction vector $\vec{U_{n}^{i}}$ on the *z* axis and is equivalent to the angle between the *y* axis and the projection of this vector on the *x*–*y* plane.

In Figure 9, the angles are represented about the unit direction vector. The verification of the orientation measures was performed using a predetermined reference. The position direction vector n at frame i is denoted by the vector $U_{n}^{i}={\left(U_{n}^{i}x,\;U_{n}^{i}y,\;U_{n}^{i}z\;\right)}^{T}\in {\mathbb{R}}^{3}$, for $i=1,\;\ldots \;,\;V_{n}$ e n=1, …, N. It is denoted by an angular vector $\vec{A_{n}}=\left(\theta_{n},\;\psi_{n},\;\phi_{n}\right)$; the vector composed of the mean of the angles measured in the n is expressed by (1).

(1) $$\vec{A_{n}}=\frac{1}{V_{n}}\sum_{i=1}^{V_{n}}\left(\theta_{n}^{i},\;\psi_{n}^{i},\;\phi_{n}^{i}\right),$$

Included in this set of angular vectors is the vector that will be used as a reference for the other N−1 positions, denoted by $\vec{A_{R}}={\left(\theta_{R},\;\psi_{R},\;\phi_{R}\;\right)}^{T}\in {\mathbb{R}}^{3}$, for $i=1,\;\ldots ,\;V_{R}$.

The angular vector of position *n* relative to the reference position is expressed by (2).

(2) $$\vec{A_{n,R}}=\vec{A_{n}}-\vec{A_{R}},$$

The actual angular displacement $A_{n,R}^{*}={\left(\theta_{n,R}^{*},\;\psi_{n,R}^{*},\;\phi_{n,R}^{*}\;\right)}^{T}\in {\mathbb{R}}^{3}$ between the reference position and the *n* position is known. It is pertinent to anticipate that, in the orientation evaluation tests, the angular displacements will be performed around each axis separately, that is, there will be no combination of two or more angles. Thus, the calculation of orientation measurement errors $E\theta_{n}$ was performed independently through (3) and analogous to $E\psi_{n}$ and $E\phi_{n}$.

(3) $$E\theta_{n}=\theta_{n,R}-\theta_{n,R}^{*},$$

**Figure 10:**
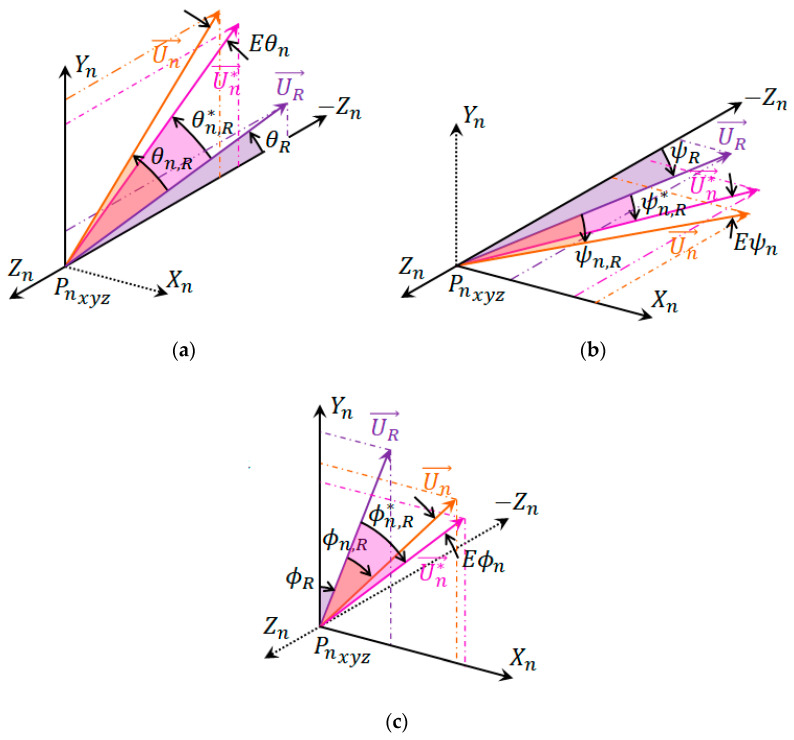
Representation of angles measured and their associated errors: (**a**) angle θ, (**b**) angle ψ, and (**c**) angle ϕ.

The angles measured in each plane, as well as the unit vectors that represent them and their associated errors, are depicted in Figure 10.

Repeatability represents the average correlation, or the degree of agreement, between successive measurements from the same orientation.

According to the standard ISO 9283 [27], the calculus of the repeatability measure $R\theta_{n}$ of each orientation n is given by (4), similar to ψ and ϕ.

(4) $$R\theta_{n}=l\theta_{n}+3S\theta_{n},$$

As there was no combination of the angles measured on the orthogonal axes, (4) was fragmented, as shown, in (5) and (6), analogous to ψ and ϕ.

(5) $$l\theta_{n}^{i}=\sqrt{{\left(\theta_{n}^{i}-\theta_{n}\right)}^{2}}=\left|\theta_{n}^{i}-\theta_{n}\right|,$$

(6) $$l\theta_{n}=\frac{1}{V_{n}}\sum_{i=1}^{V_{n}}\left|\theta_{n}^{i}-\theta_{n}\right|,$$

and the standard deviation of the measured angles, as expressed in (7), analogous to ψ and ϕ.

(7) $$S\theta_{n}=\sqrt{\frac{1}{V_{n}-1}\sum_{i=1}^{V_{n}}{\left(l\theta_{n}-l\theta_{n}^{i}\right)}^{2}},$$

### 4.2. Angular Displacement Experiment

**Figure 11:**
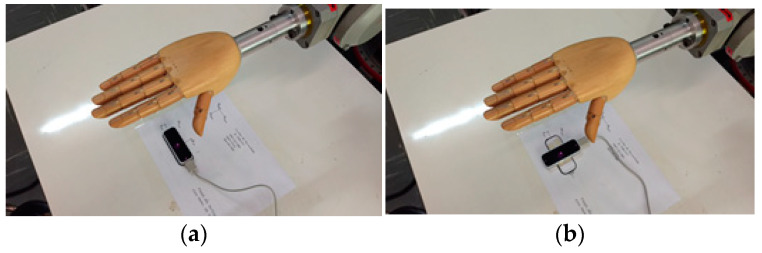
LMC configurations used in the experiments: (**a**) Transversal and (**b**) Longitudinal.

During the elaboration of the experiments, we considered which would be the best orientation of the LMC about the wooden hand and which would result in better quality in the readings. Thus, two configurations were verified in each type of experiment: a transversal configuration, whose axis parallel to the hand is the transversal axis of the LMC, recommended for use by the manufacturer, Figure 11a, and a longitudinal configuration, whose axis parallel to the hand is the longitudinal axis of the LMC, Figure 11b.

For the angular displacement experiments, the average of the α angle, referring to the hand opening angle, was also calculated, Figure 7. Since the calculation of the α angle by the API is performed from the vector of the average direction of the fingers, the error was not calculated about the measurements made by the goniometer. It was observed, instead, if the sensor could identify the different hand configurations and the standard deviation of these measurements. The angular displacement experiments have the following specific objectives: the observation of the LMC acquisition rate; the verification of the consistency of the readings made when there is a change in the orientation of the hand; the calculation of the error of angular measurements; the repeatability assessment in each considered position; the verification of the best angles for measuring angular positions; the verification of the quality of the readings for different configurations of the wooden hand, Figure 7; and the determination of the opening angles for each hand configuration.

**Figure 12:**
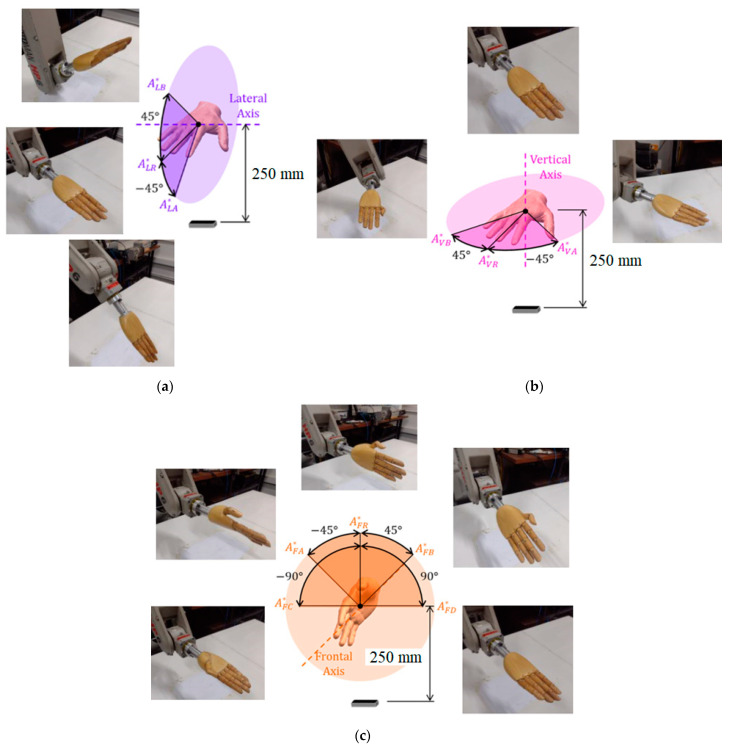
Angular displacement experiments on the (**a**) lateral, (**b**) vertical, and (**c**) front axes.

There are three angular displacement experiments, each rotating along the lateral, vertical, and frontal axes. On the vertical and lateral axes, two angular positions ‘A’ and ‘B’ were measured from the reference ‘R’, oriented 45° in both directions of the axis, and the reference orientation remained with the palm pointing downwards. On the front axis, there are four angular positions ‘A’, ‘B’, ‘C’, and ‘D’, and the reference is oriented with the thumb pointing upwards, Figure 12.

The simulations exactly replicate the hand and wrist motions that are performed by the robot in the experiments, such as those shown in Figure 12. Eighteen angular displacement tests were made to reach and measure a total of forty-eight angular positions that were evaluated in total, since each test reaches three hand configurations, as shown in Figure 7, for each of the three axes, while two LMC configurations are tested for each case.

## 5. Experimental Results

**Figure 13:**
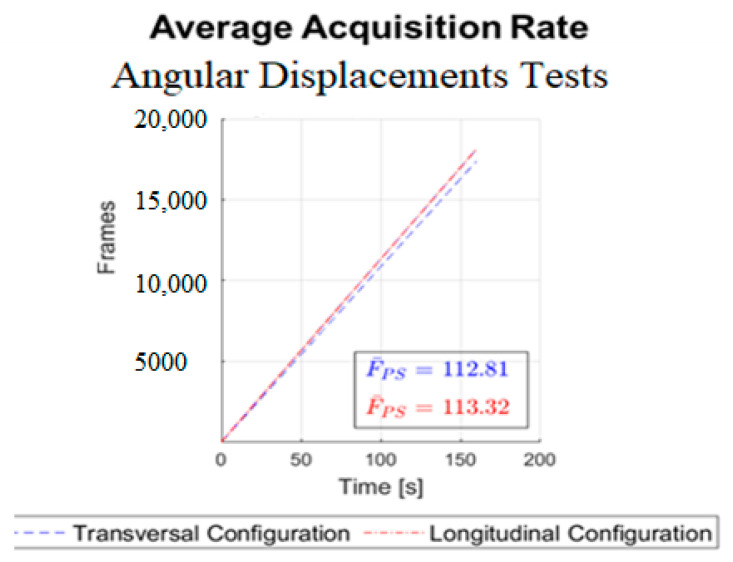
Mean acquisition rates.

Before starting the experimental tests, some procedures were taken, such as closing the curtains, reducing the interference of infrared external light. The API has some options disabled that can impact the experimental results such as automatic orients of the axis and the robust mode that increases in some moments the infrared LEDs intensity. It is necessary to point out that the experiments were conducted using a wooden hand coupled with a metal rod, which are reflective surfaces. In general, reflective surfaces can affect the quality of the measurements. This can be particularly relevant when using specific LMC settings and lighting conditions. Accordingly, several attempts were made to verify the influence of the lighting conditions. Furthermore, it has been useful to disable the “robust mode” set up of the LMC sensors, since this option varies the infrared LEDs’ intensity and negatively affects the uniformity of the collected data. The mean acquisition rate in frames per second of all experiments and sensor configuration was verified, Figure 13.

From Figure 13, the shape of the curve is a straight line, which represents a constant acquisition rate. The average rate near 113 fps is extremely high compared to the range of 15 to 30 fps considered satisfactory for application in games [28]. The calculated mean acquisition rates were three times that observed by [16] and about double that observed by [29]. In [16,29], the SDK version 1 was used, and version 3 has been improved in this respect.

**Figure 14:**
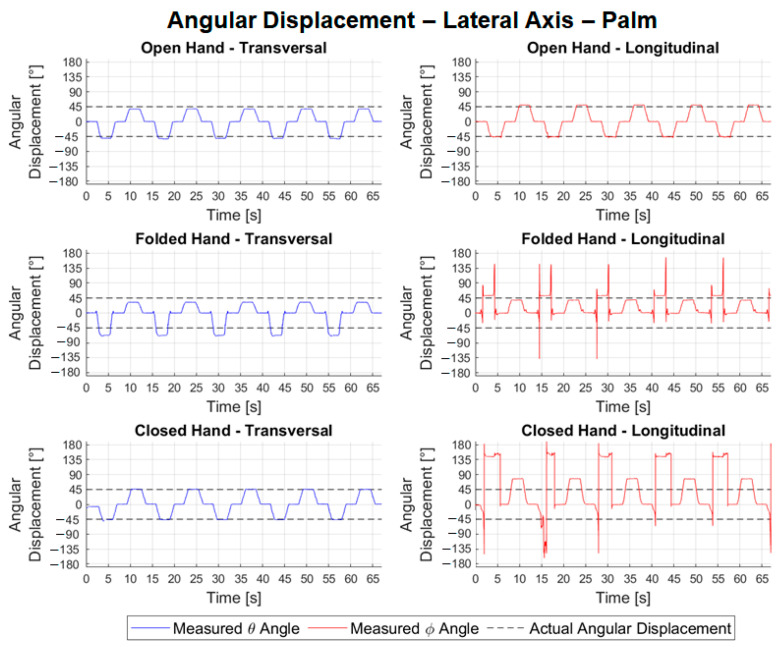
Relative angular positions of the palm to the lateral angular displacement.

**Figure 15:**
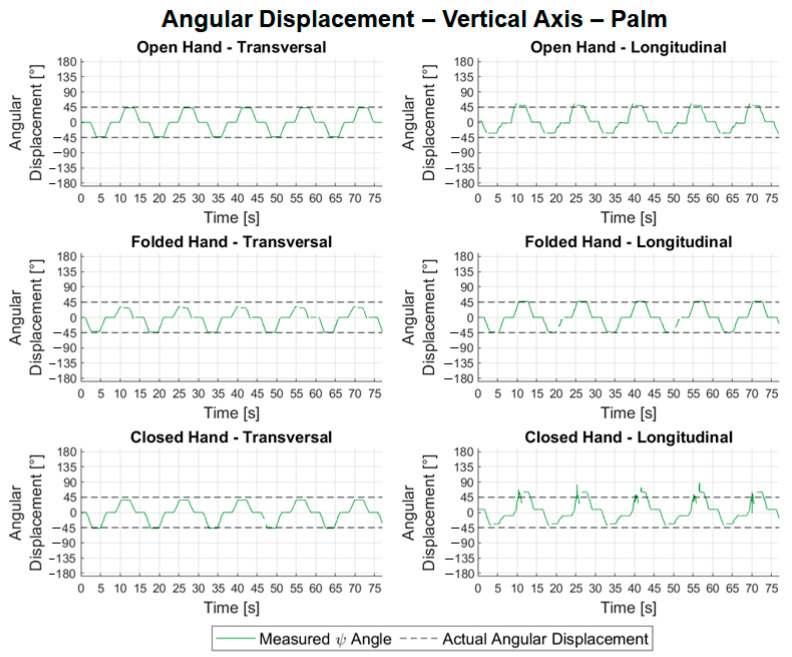
Relative angular positions of the palm to the vertical angular displacement.

In Figure 14, for lateral angular displacements, the waveforms observed in the transversal configuration tests are consistent with the angular displacements performed by the hand, whereas in the longitudinal configuration tests, only the graph referring to the open hand shows a suitable waveform. In the graphs corresponding to the longitudinal configuration tests with the folded and closed hands, a phenomenon stands out in a 90° and 180° inversion, respectively, in the predominant axis when the closed hand is oriented at −45° and the sensor reads angle *ϕ* close to 45° and 135°, respectively. For the vertical angular displacements shown in Figure 15, where the predominant axis is the *y* axis, then the angle *ψ* (in green) is measured. The waveform observed in all experiments is consistent with the trajectories performed; however, both in the transversal experiment with the folded hand and in the longitudinal ones, there is a certain discontinuity close to the 45° angle.

**Figure 16:**
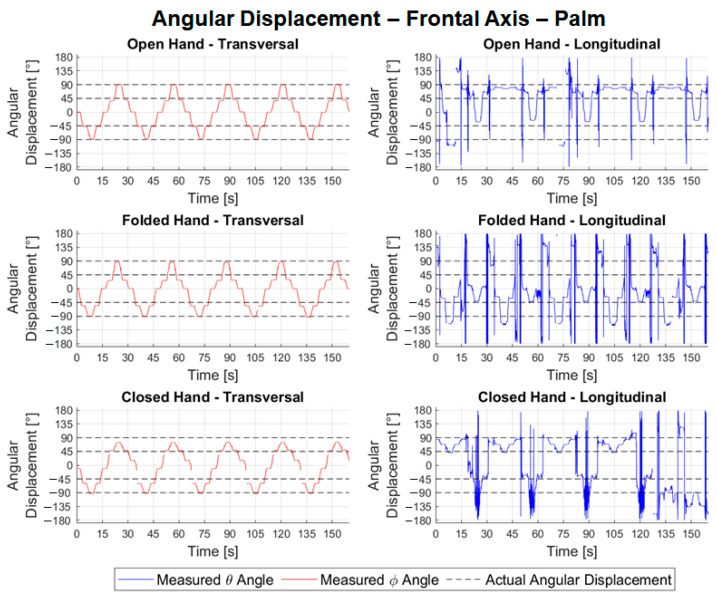
Relative angular positions of the palm to the frontal angular movement.

In Figure 16, the predominant axis of the transversal frontal movements is the *z* axis (corresponding to the angle *ϕ*, in red), and, in the longitudinal ones, it is the *x* axis (corresponding to the angle *θ* in blue). The tests carried out with the transversal configuration have a good waveform, being consistent with the trajectory performed by the wooden hand. Longitudinal experiments have a very noisy waveform, making it impossible to identify positions for error and repeatability calculations.

Table 1 shows the results of the angular movements. Table 2 shows the absolute values of the errors and mean repeatability of the angular movement tests for the different hand configurations.

Analyzing the results Table 1, we noted that the consistency of all lateral angular measurements was close to 100%, characterizing an excellent quality of the samples. For transversal configuration, the average error calculated for variable *θ* was −8.13° with a maximum absolute error of 22.65°. In the longitudinal configuration tests, the mean errors were 53.30°, with a maximum of 167.62°. It is believed that the high error value in the longitudinal configuration is due to the inversion phenomenon observed in Figure 14. The problem of the inversion phenomenon or the possible occlusion can be decreased by the use of two LMC such as those proposed in [30]. The mean repeatability for the lateral tests in both configurations, however, remained low, with values of 1.74° and 2.16° for the transversal and longitudinal tests, respectively. It is also noteworthy that it is possible to compare the errors calculated in the open hand of 6.33°, Table 2, in the transversal configuration (lateral axis) with the error observed by [20], in the flexion/extension experiments (RMS error of 11.6°). It is noted that, in the module, the calculated error was less than the observed error. In Table 1, the data for the angular movement around the vertical axis showed the average percentage of valid frames was 97.37% for transversal configuration tests and 92.90% for longitudinal. The average of errors in the transversal experiments was −4.07°, with an absolute maximum of 15.88°, and in the longitudinal experiments, it was 8.12°, with a maximum of 15.52°. It is also possible to compare the errors calculated for the open hand with those measured in the abduction/adduction experiments by [20] (RMS error of 12.4°). In the case of the open hand, in the transversal configuration, the error found was much less than 2.09°.

The repeatability in the readings of the transversal tests related to the movement around the vertical axis was 0.85°, lower than in the longitudinal ones, 2.65°, Table 1. From Table 1, for the frontal tests, it is possible to observe good consistency in the readings, representing an average of 98.94% of frames valid for transversal configuration experiments and 98.36% for longitudinal ones. The average of the errors for the angular movements around the frontal axis in the transversal tests was −5.56°, with an absolute maximum of 17.04°, Table 3. Regarding the longitudinal experiments, due to the observed noise, it was not possible to calculate the errors in each angular positioning. The repeatability associated with the transversal configuration tests had an average value of 2.70° with a maximum of 20.61°. In the longitudinal test, it was not possible to perform the calculation.

It is also possible to verify the absolute maximum error of 6.33°, Table 2, found for the open hand about that observed in the supination/pronation experiment by [20] of 38.4°. In general, all axes obtained small errors in the experiments with angular movements in the transversal configuration, Table 1. It is possible to observe, in Table 2, that the absolute values of the average errors for the angles *ψ* and *ϕ* were lower for the open hand, while the angle *θ* was smaller for the closed hand. Regarding the observed average repeatability, the open and folded hand configurations generally presented lower values than the closed hand. Table 3 shows the mean values $\bar{\alpha }$ and deviations 𝑆𝛼 of the hand opening angles for each of the angular movement tests, Figure 8. In Table 3, the deviations of the opening angle for the tests of transversal angular movements were smaller than for the longitudinal ones. It can also be seen that the sensor can identify the different configurations of the hand by distinguishing between the angles. Goniometry is widely used for the measurement of ROM in clinical practice. For each joint, diverse types of goniometers are required. Despite the manual goniometer being low-cost, portable, and lightweight, the made measures have problems in the functions of the experience of physiotherapists [31,32,33].

The manual goniometer permits only statical measurements and individual joint measurements. From the experimental results, Figure 14, Figure 15 and Figure 16, the LMC can be used to track the ROM continuously and used in the development of serious games. In [34], the LMC was used to calculate and represent graphically static and dynamic hand parameters of the ROM in a healthy subject. In [35], the LMC was used to measure the wrist ROM compared to the goniometry obtained good results except for ulnar/radial deviation in healthy subjects. As highlighted in [35], it is necessary to make tests with pathological populations. This gives the motivation for further investigations, as proposed in this work, to consider the use of LMC for the requirements and constraints of a specific rehabilitation treatment. The main novelty of this work consists of addressing the use of LMC when treating some specific pathology configurations. Despite the LMC being used for more than nine years with several applications for rehabilitation as presented in [6,7,8,9,10,11,12,13], the experimental tests with patients still show several limitations of the LMC. This paper contributes to the simulation of pathologies to guide future research with patients. In terms of the function of the different hand configurations necessary in the rehabilitation procedures, the open and closed configuration to the different wrist movements gives acceptable values and permits the development of serious games.

Based on the analyses made from the described experiments, some conclusions can be drawn regarding its use in the development of serious game/rehabilitation movements when applied to wrist rehabilitation. The first conclusion is that the sensor acquisition rate can be suitable for the application in clinical/hospital close environments where the interference of external light can be minimized. Indeed, the measured acquisition rate is higher that the requirements for integration in serious games. Analyzing the angular displacement experiments, the best option for the configuration of the LMC is the transversal arrangement that managed to track the hands during the orientation change in the three-axes wrist. The angles results presented in this paper were calculated by LMC automatically. It is important to point out that the longitudinal configuration is not the recommended default setup to use the LMC device, and the user needs to guarantee the correct orientation of the device to have valid measurements. The LMC can be used to continuously track the wrist’s range of motion to be used in the clinical evolution of patient’s rehabilitation. The LMC can identify if the fingers are opened, folded, or closed. In terms of the fingers’ orientation, the LMC can properly identify with small errors the wrist movements when opened and closed. In the folded configurations, the flexion errors are considerable.

## 6. Conclusions

This paper has addressed the analysis and validation of using a Leap Motion Controller (LMC) in wrist-rehabilitation tasks. The proposed LMC performance analysis has been made through the analysis of its positioning error, repeatability, and acquisition rate. Particular attention has been paid to the measurement and analysis of the angular configuration of the hand, which is fundamental for wrist rehabilitation and has never been addressed in previous studies on LMC. To the best of our knowledge, this paper is the first to evaluate the angular measurement’s movements with a different configuration of the hand. The results were obtained with the use of a wooden hand fixed on an industrial robotic arm. The industrial robot simulates the angular poses of a human wrist while providing an accurate measurement of such poses that can be compared with the measurements obtained via the LMC. The obtained experimental results show the feasibility of the LMC as a suitable low-cost option for wrist rehabilitation. Further experimental tests with humans will be planned as future steps of this research, by integrating serious game strategies together with a LMC to further verify the effectiveness of the device in the wrist rehabilitation considering the specific characteristics and limitations on the measurements of the hand’s angular configurations.

## Figures and Tables

**Table 1:** Mean errors, repeatability, and consistency for the tests of angular movements.

Transversal Configuration			
Axis	$\bar{E_{R}}\left[{}^{\circ}\right]$	$\bar{R_{n}}\left[{}^{\circ}\right]$	$\bar{V}\left[\%\right]$
Lateral	−8.13	1.74	99.75
Vertical	−4.07	0.85	97.37
Frontal	−5.56	2.70	98.94
Max.	22.65	20.61	-
Longitudinal Configuration			
Axis	$\bar{E_{R}}\left[{}^{\circ}\right]$	$\bar{R_{n}}\left[{}^{\circ}\right]$	$\bar{V}\left[\%\right]$
Lateral	53.30	2.16	100.00
Vertical	8.12	2.65	92.90
Frontal	-	-	98.36
Max.	167.62	8.75	-

**Table 2:** Absolute values of angular errors and average repeatability for different hand configurations.

Transversal Configuration						
Axis	Lateral		Vertical		Frontal	
Hand	$\bar{E\theta_{R}}\left[{}^{\circ}\right]$	$\bar{R\theta_{n}}\left[{}^{\circ}\right]$	$\bar{E\psi_{R}}\left[{}^{\circ}\right]$	$\bar{R\psi_{n}}\left[{}^{\circ}\right]$	$\bar{E\phi_{R}}\left[{}^{\circ}\right]$	$\bar{R\phi_{n}}\left[{}^{\circ}\right]$
Open	6.33	1.12	2.09	0.33	3.02	1.27
Folded	17.63	0.96	8.73	2.05	8.28	1.94
Closed	1.05	3.14	5.10	0.17	9.36	4.88
Longitudinal Configuration						
Axis	Frontal		Vertical		Lateral	
Hand	$\bar{E\theta_{R}}\left[{}^{\circ}\right]$	$\bar{R\theta_{n}}\left[{}^{\circ}\right]$	$\bar{E\psi_{R}}\left[{}^{\circ}\right]$	$\bar{R\psi_{n}}\left[{}^{\circ}\right]$	$\bar{E\phi_{R}}\left[{}^{\circ}\right]$	$\bar{R\phi_{n}}\left[{}^{\circ}\right]$
Open	-	-	8.99	1.82	2.10	1.19
Folded	-	-	2.35	0.43	45.44	1.26
Closed	-	-	13.01	5.70	112.36	4.04

**Table 3:** Values of hand opening to angular movements.

Transversal Configuration						
Hand	Open		Folded		Closed	
Axis	$\bar{\alpha }\left[{}^{\circ}\right]$	$\bar{S_{\alpha }}\left[{}^{\circ}\right]$	$\bar{\alpha }\left[{}^{\circ}\right]$	$\bar{S_{\alpha }}\left[{}^{\circ}\right]$	$\bar{\alpha }\left[{}^{\circ}\right]$	$\bar{S_{\alpha }}\left[{}^{\circ}\right]$
Lateral	17.22	8.09	105.91	20.67	176.91	7.93
Vertical	23.18	6.58	132.61	21.03	180.00	0.02
Frontal	10.89	7.59	150.30	23.43	175.99	10.40
Mean	17.09	7.42	129.61	21.71	177.63	6.12
Longitudinal Configuration						
Hand	Open		Folded		Closed	
Axis	$\bar{\alpha }\left[{}^{\circ}\right]$	$\bar{S_{\alpha }}\left[{}^{\circ}\right]$	$\bar{\alpha }\left[{}^{\circ}\right]$	$\bar{S_{\alpha }}\left[{}^{\circ}\right]$	$\bar{\alpha }\left[{}^{\circ}\right]$	$\bar{S_{\alpha }}\left[{}^{\circ}\right]$
Lateral	33.25	14.23	99.38	41.18	160.82	14.07
Vertical	44.30	11.79	103.30	10.38	157.04	20.15
Frontal	45.50	27.99	130.98	28.57	141.28	53.98
Mean	41.02	18.00	111.22	26.71	153.04	29.40

## Data Availability

Not applicable.
